# Supplementary material for: Multi‐dataset identification of innovative feature genes and molecular mechanisms in keratoconus
Source: J Cell Mol Med. 2024 Sep 19;28(18):e70079. doi: 10.1111/jcmm.70079 (PMC11412914; doi:10.1111/jcmm.70079)
Supplement: Supplementary file 2 — Material S2. [file JCMM-28-e70079-s002.zip › 7.GSEA analysis/4.MYRF/KEGG_GLYCOSYLPHOSPHATIDYLINOSITOL_GPI_ANCHOR_BIOSYNTHESIS.html]

Details for gene set KEGG\_GLYCOSYLPHOSPHATIDYLINOSITOL\_GPI\_ANCHOR\_BIOSYNTHESIS[GSEA]

|  || Dataset | MYRF.group.cls#h\_versus\_l |
| Phenotype | group.cls#h\_versus\_l |
| Upregulated in class | h |
| GeneSet | KEGG\_GLYCOSYLPHOSPHATIDYLINOSITOL\_GPI\_ANCHOR\_BIOSYNTHESIS |
| Enrichment Score (ES) | 0.16747342 |
| Normalized Enrichment Score (NES) | 0.5182176 |
| Nominal p-value | 0.95573443 |
| FDR q-value | 1.0 |
| FWER p-Value | 1.0 |
Table: GSEA Results Summary

  

Fig 1: Enrichment plot: KEGG\_GLYCOSYLPHOSPHATIDYLINOSITOL\_GPI\_ANCHOR\_BIOSYNTHESIS      
 Profile of the Running ES Score & Positions of GeneSet Members on the Rank Ordered List

  

| SYMBOL | TITLE | RANK IN GENE LIST | RANK METRIC SCORE | RUNNING ES | CORE ENRICHMENT || 1 | PGAP1 | na | 3072 | 0.291 | 0.0849 | Yes |
| 2 | PIGM | na | 6343 | 0.182 | 0.0991 | Yes |
| 3 | PIGL | na | 7304 | 0.156 | 0.1642 | Yes |
| 4 | PIGW | na | 12070 | 0.063 | 0.0642 | Yes |
| 5 | PIGY | na | 12631 | 0.055 | 0.0807 | Yes |
| 6 | PIGX | na | 15065 | 0.027 | 0.0269 | Yes |
| 7 | GPAA1 | na | 15536 | 0.022 | 0.0267 | Yes |
| 8 | PIGS | na | 16601 | 0.012 | 0.0032 | Yes |
| 9 | PIGC | na | 17165 | 0.007 | -0.0087 | Yes |
| 10 | PIGN | na | 17406 | 0.005 | -0.0127 | Yes |
| 11 | PIGK | na | 17455 | 0.005 | -0.0114 | Yes |
| 12 | PIGQ | na | 18971 | -0.010 | -0.0491 | Yes |
| 13 | GPLD1 | na | 19116 | -0.012 | -0.0462 | Yes |
| 14 | PIGG | na | 19632 | -0.017 | -0.0506 | Yes |
| 15 | PIGF | na | 20876 | -0.032 | -0.0674 | Yes |
| 16 | PIGO | na | 20929 | -0.033 | -0.0495 | Yes |
| 17 | PIGT | na | 22204 | -0.048 | -0.0574 | Yes |
| 18 | PIGH | na | 22266 | -0.049 | -0.0299 | Yes |
| 19 | PIGP | na | 22462 | -0.052 | -0.0048 | Yes |
| 20 | PIGZ | na | 22714 | -0.055 | 0.0208 | Yes |
| 21 | PIGU | na | 23073 | -0.061 | 0.0467 | Yes |
| 22 | PIGV | na | 23919 | -0.074 | 0.0665 | Yes |
| 23 | DPM2 | na | 24410 | -0.082 | 0.1015 | Yes |
| 24 | PIGB | na | 27233 | -0.140 | 0.1038 | Yes |
| 25 | PIGA | na | 28916 | -0.188 | 0.1675 | Yes |
Table: GSEA details [plain text format]

  

Fig 2: KEGG\_GLYCOSYLPHOSPHATIDYLINOSITOL\_GPI\_ANCHOR\_BIOSYNTHESIS      
 Blue-Pink O' Gram in the Space of the Analyzed GeneSet

  

Fig 3: KEGG\_GLYCOSYLPHOSPHATIDYLINOSITOL\_GPI\_ANCHOR\_BIOSYNTHESIS: Random ES distribution      
 Gene set null distribution of ES for **KEGG\_GLYCOSYLPHOSPHATIDYLINOSITOL\_GPI\_ANCHOR\_BIOSYNTHESIS**
